# Depletion of donor-specific anti-HLA A2 alloantibodies in a hematopoietic cell transplant recipient using directed mismatched platelet transfusions

**DOI:** 10.1038/s41409-018-0220-7

**Published:** 2018-05-24

**Authors:** Bernd M. Spriewald, Christian Bach, Juergen Zingsem, Julian Strobel, Julia Winkler, Andreas Mackensen, Wolf Roesler

**Affiliations:** 1 Department of Internal Medicine 5, Hematology/Oncology, University Hospital Erlangen, Erlangen, Germany; 2 Department of Transfusion Medicine and Hemostaseology, University Hospital Erlangen, Erlangen, Germany

Donor-specific anti-HLA alloantibodies (DSA) are a risk factor for graft failure in HLA-mismatched allogeneic hematopoietic cell transplantation [1–4]. This seems to be particularly true when DSA can be detected at a high level in solid-phase assays [5], or are complement binding as detected in the C1q allo-antibody assay [6] and the complement-dependent cytotoxicity (CDC) crossmatch [7]. DSA may also have a negative impact on overall survival after reduced intensity conditioning [8]. With increasing use of HLA-mismatched stem cell donors and improved diagnostic methods for anti-HLA antibodies, this problem becomes clinically even more relevant. Reduction of DSA prior to transplantation is able to reduce the risk of graft failure and facilitates engraftment [4–6, 9]. However, it is still unclear which method is the most suitable for desensitization [10]. Here we report the successful desensitization prior to a HLA-A2 mismatched allogeneic hematopoietic cell transplantation (HCT) in a highly immunized patient, demonstrating the incremental effect of directed HLA-A2 mismatched platelet transfusions on the DSA level.

**Fig. 1:**
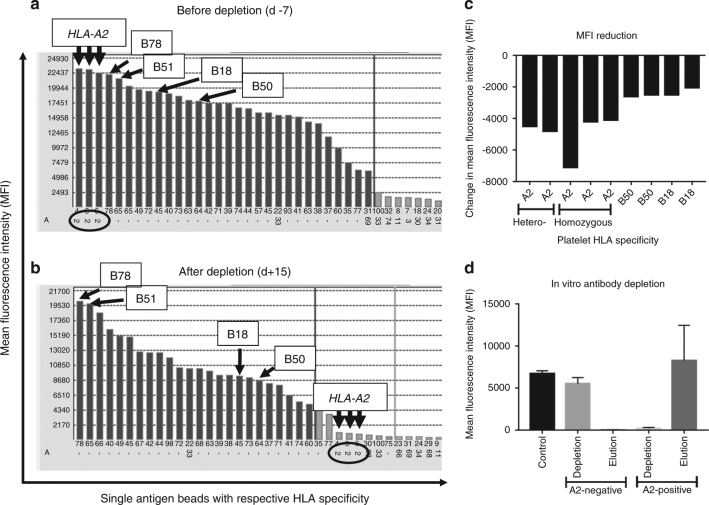
Details of the results sheet of the single antigen HLA antibody test before depletion (day −7, **a**) and after depletion (day +15, **b**) are shown. The anti-A2 antibody columns are indicated with thick black arrows. All highly positive reactions against HLA-A2 (**a**) became negative (**b**). In contrast, antibodies towards bystander HLA specificities that were co-expressed on the transfused platelets, such as HLA-B18 and B50, were only partially reduced, whereas third party specificities not expressed on the platelets, such as HLA-B78 and B51, remained at a high level. The incremental effect of each of the first five platelet transfusions and the respective antigen is shown in **c**. The two transfusions given just before the hematopoietic cell transplant were not included since the data would be skewed by the high number of A2-positive cells within the transplant. We observed a stable MFI reduction for the HLA-A2 DSA, whereas the bystander antigens of the HLA-B locus were less effective. To investigate the specificity of the HLA-A2-expressing platelets, the anti-HLA antibodies of the day −7 serum were depleted using either HLA-A2 positive (*n* = 3) or negative (*n* = 3) platelets as follows: 20 µl of recipient serum were incubated with 120 µl of a platelet suspension containing 1 × 10^9^ platelets/ml in 0.9% NaCl for 30 min at 37 °C. Subsequently, the suspension was centrifuged for 5 min at 12,000 × *g*. The supernatant was removed and depleted two more times as described, before being analyzed in the solid-phase antibody test. Serum treated with PBS was used as control. To prove that the depleted antibody was adsorbed by the platelets, the centrifuged platelets were subjected to an acid elution using a commercial elution kit according to the manufacturer’s instructions (BAG-Elutions-Kit, BAG Health Care GmbH, 35423 Lich, Germany) and the eluate was analyzed for anti-HLA antibodies. The results of three independent runs with different platelet preparations are shown in (**d**), as the mean ± s.d. Whereas depletion with HLA-A2-negative platelets had no effect on the level of the anti-A2 antibody, it disappeared after depletion with A2-positive platelets. In contrast, elution of antibodies from the respective platelets demonstrated that the anti-A2 antibody could be recovered from the A2-positive platelets but not the A2 negative. This shows that depletion of the anti HLA-A2 antibody was a specific effect of the A2 expressing platelets

Three months before transplantation, the patient, a then 53-year-old female, was diagnosed with acute myloid leukemia with an intermediate genetic risk profile. She received a standard 7 + 3 induction therapy with cytarabine and daunorubicin. Bone marrow control puncture on day 14 revealed induction failure and salvage therapy consisting of high-dose cytarabine and mitoxantrone was applied. In addition, the search for an allogeneic stem cell donor was initiated. The best available donor identified was a 9/10 matched unrelated donor, with the donor carrying HLA-A2 as the single antigen mismatch for HLA-A, -B, -C, -DRB1 and -DQB1. In addition, one permissive antigen mismatch for HLA DPB1 was found. During aplasia following induction therapy, the patient became refractory to platelet transfusions. Broad immunization towards HLA class I was confirmed when the patient’s serum was analyzed for HLA antibodies using a single antigen solid-phase assay (Labscreen, One Lambda, Thermo Fisher Scientific, Canoga Park, CA, USA). All sera were used undiluted and subjected to heat inactivation and filtration (AcroPrep Advance 96 Filter Plate, PALL Life Sciences, Ann Arbor, MI, USA) before being analyzed in order to reduce the so called prozone effect [11]. No antibodies against HLA-DP were present. Antibodies directed towards HLA-A2, which was also part of the paternal haplotype inherited by the patient’s two children, showed the highest mean fluorescence intensity (MFI, Fig. 1a). In contrast to ABO-isoagglutinins, which are determined by titration, the level of anti-HLA antibodies in single antigen bead assays is reported by the mean fluorescence intensity, although recent studies suggest that serial dilution of sera before subjecting them to solid-phase assays may more accurately determine antibody strength in these assays [12]. The patient’s serum also reacted strongly with lymphocytes from the selected stem cell donor in a complement-dependent cytotoxicity crossmatch (CDC XM), providing the strongest evidence for the clinical relevance of the DSA. Due to the high level of DSA and the positive CDC crossmatch, desensitization was initiated prior to HSCT, using directed HLA-A2 mismatched platelet transfusions in combination with rituximab and bortezomib.

**Fig. 2:**
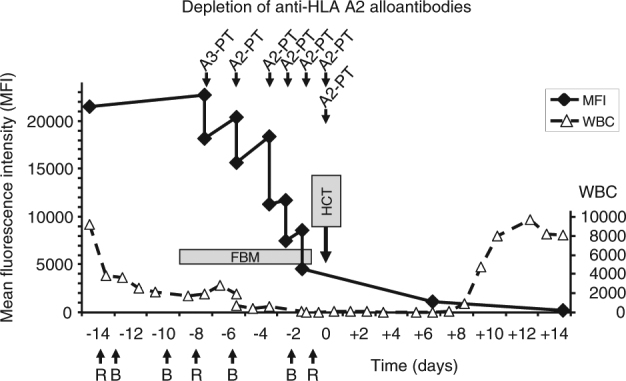
The mean fluorescence intensity (MFI) of the HLA-A2 antibody is depicted in black diamonds. White blood cell count is shown as open triangles. The time points of HLA-A2-positive apheresis platelet transfusion (A2-PT) are indicated as well as the application of rituximab (R) and bortezomib (B). FBM = non-myeloablative conditioning with fludarabine, carmustine, melphalan and antithymocyte globulin; HCT = hematopoietic cell transplantation on day zero

Reduced intensity conditioning with fludarabine, carmustine, melphalan, and antithymocyte globulin was started on day −10 pre-transplant. Between days −14 and −1, the patient received four courses of rituximab (375 mg/m²) and four courses of bortezomib (1.3 mg/m²). In addition, a total of seven HLA-A2-positive platelet apheresis products were transfused, on days −7, −5, −3, −2, −1 and two on day 0, just prior to the infusion of 6.6 × 10^6^ CD34+/kg unmanipulated peripheral blood stem cells. Anti-HLA antibodies were measured before and 2–3 h after each directed platelet transfusion until day −1, and on day +7 and +15. The desensitization treatment and DSA levels are summarized in Fig. 2. Since therapeutic antibodies such as rituximab interfere with the CDC crossmatch, we performed a Luminex® crossmatch (Lifecodes DSA Detection, Immucor Inc., Norcross, GA, USA). The crossmatch with serum from day −7 was positive, whereas with serum from day −1, which had a residual DSA of MFI 4500, the test was already negative. The DSA reduction continued after transplantation most likely reflecting adsorbtion of the antibody by engrafting donor cells (Fig. 2). On day +15, the anti-HLA A2 antibody was barely detectable in the single antigen bead assay (Fig. 1b). The patient engrafted on day 10 and the last platelet transfusion was required on day +7. GvHD prophylaxis was based on cyclosporine and mycophenolate mofetil. Apart from a short episode of grade II acute GvHD of the upper gastrointestinal tract, the follow-up was uneventful. The patient remained in complete remission over 1 year after transplantation, and chimerism analysis continued at 100% donor from the first control on day 30 onwards.

In our patient the donor-specific antibody was directed against a paternal HLA antigen inherited by both her two children. This is consistent with the previous data showing the highest incidence of donor-specific anti-HLA antibodies of up to 40% in female patients with prior pregnancies [9]. Although there are no defined thresholds that distinguish a prohibitive from a permissive anti-HLA donor-specific antibody, the current data indicate that a high MFI value > 10,000 and complement binding of the DSA constitute risk factors for graft failure [5, 6]. If there is no alternative stem cell donor available in order to avoid the respective HLA mismatches, desensitization can be performed to reduce the DSA level. Various methods for desensitization have been described, including mainly plasmapheresis supported by high-dose intravenous immunoglobulin, rituximab or bortezomib. Also transfusions of platelets or irradiated donor lymphocytes, with or without combined plasmapheresis, have been applied [10, 13]. However, the most effective approach is not yet defined. The data obtained from solid organ transplantation show that the various desensitization protocols show different levels of efficiency, with immunoadsorption seemingly being the most effective, and they also show that a high burden of DSA may not completely be removed before transplantation [14]. Whereas all the methods for desensitization used in solid organ transplantation, including plasmapheresis, immunoadsorption, high-dose intravenous immunoglobulin and rituximab could be applied in hematopoietic cell transplantation, the use of platelet transfusion to remove anti-HLA class I antibodies is unique to stem cell transplantation.

Here we decided to use directed mismatched platelet transfusions without additional plasmapheresis. Platelets express HLA class I but not class II antigens on their cell surface [15], and can be used to deplete antibodies against HLA class I antigens in vitro [16], and also in vivo [5, 17]. For desensitization, seven apheresis platelet preparations with an average platelet content of 3.2 × 10^11^ platelets per unit were transfused. Importantly, the patient did not experience any transfusion reaction. The first two platelet preparations came from HLA-A2 heterozygous and the following five from A2 homozygous donors. However, this did not seem to have a major effect on the antibody reduction capacity, since each platelet transfusion reduced the MFI of the DSA by an average of nearly 5000 units (Fig. 1c and 2). Notably, all but one of the platelet productsused for DSA depletion had additional HLA class I antigens against which the recipient was immunized. Comparison of the antibody profile on day −7 and + 15 (Fig. 1a,b) shows that the anti HLA-A2 DSA was strongly reduced. In contrast, antibodies towards HLA specificities expressed on the transfused platelets as bystander antigens (HLA-B18 and B50) decreased only partially, whereas third party specificities not expressed on the platelets remained at a high MFI level (e.g., HLA-B78 and B51, Fig. 1a–c). The less effective depletion of the bystander antibodies directed towards HLA-B18 and B50 was partially due to the fact that platelets with these antigens were transfused only twice for each of the respective antigens. However, we also noted that the depletion capacity of a single dose of B antigens was weaker compared to the A2 antigen (Fig. 1c). Whether this is just a coincidence observed in this case study, or reflects a more general phenomenon, either of a differential alloantibody binding capacity or of a differential expression of various HLA antigens or loci, has to be addressed in further studies. The persisting antibodies not depleted by the platelet transfusion indicate that the conditioning regime as well as rituximab and bortezomib, had only a limited effect on the anti-HLA antibody levels in this protocol. Since there is no generally accepted guideline how to desensitize a recipient with DSA, we had included rituximab and bortezomib in the pretreatment following protocols used in solid organ transplantation. In the context of our case, bortezomib might help to delay a recurrence of the DSA, although there are no current data to support this concept. It was also interesting to note that, despite a very high initial mean fluorescence intensity (MFI > 20,000), seven directed platelet transfusions were sufficient to strongly reduce the DSA. Whether directed platelet transfusions will be sufficient to deplete anti-HLA antibodies in such a strong way to allow engraftment in all cases cannot be decided from the currently available data. After HCT, the patient became anti HLA-A2 negative and the antibody was undetectable during the follow-up of over 1 year (data not shown). Whether a combination of platelet transfusion with therapeutics such as rituximab or bortezomib is having an additional effect, or whether platelets in combination with the conditioning regimen alone are sufficient to allow long-term engraftment remains to be seen. The initial reduction of the anti HLA-A2 DSA was a specific effect of the A2 expressing platelets. This was shown in further in vitro experiments using HLA-A2-positive and -negative platelet preparations for depletion and elution of the anti HLA-A2 antibody (Fig. 1d).

In summary, this case shows that directed HLA-mismatched platelet transfusion is feasible and can result in a strong, dose-dependent reduction of anti-HLA antibodies. However, since platelets express only HLA class I antigens on their cell surface, it has to be pointed out that their use in desensitization is only reasonable in patients with HLA antibodies directed against HLA class I donor antigens. Since HLA antibody depletion is specific for the respective HLA antigen, platelets expressing the corresponding mismatched antigen have to be used. However, we have to point out that these data are derived from a single case report, and since there are no untreated controls, we cannot provide evidence of a direct impact of the DSA reduction on the transplant itself.
